# Comparison of Caffeine versus Theophylline for apnea of prematurity

**DOI:** 10.12669/pjms.35.1.94

**Published:** 2019

**Authors:** Arif Zulqarnain, Muddasser Hussain, Khalid Munir Suleri, Zafar Ali Ch.

**Affiliations:** 1 Dr. Arif Zulqarnain, MBBS FCPS. Department of Pediatrics, Medicine Govt. Khawaja Muhammad Safdar Medical College & Govt. Allama Iqbal Memorial Teaching Hospital, Sialkot, Pakistan; 2 Dr. Muddasser Hussain, MBBS, FCPS. Department of Pediatrics, Medicine Govt. Khawaja Muhammad Safdar Medical College & Govt. Allama Iqbal Memorial Teaching Hospital, Sialkot, Pakistan; 3 Dr. Khalid MunirSuleri, MBBS, BSC, DCH. Department of Pediatrics, Suleri Children and General Hospital, Sialkot, Pakistan; 4 Dr. Zafar Ali Ch., MBBS, FCPS, FRCS. Department of Pediatrics, Medicine Govt. Khawaja Muhammad Safdar Medical College & Govt. Allama Iqbal Memorial Teaching Hospital, Sialkot, Pakistan

**Keywords:** Caffeine, Theophylline, Apnea, Infants, Prematurity

## Abstract

**Objective:**

To make a comparison between standard doses of theophylline and caffeine for the treatment of apnea of prematurity.

**Methods:**

A randomised control trail was conducted in Department of Pediatrics Medicine Govt. Khawaja Muhammad Safdar Medical College and Govt. Allama Iqbal Memorial Teaching Hospital, Sialkot over duration of one year from August 2017 to August 2018 after approval from the hospital ethics committee. An informed consent in the form of written document was also taken from the parents of each infant participating in the study. Data was analyzed by using SPSS version 24. Mean and SD was calculated for numerical data like gestational age and mean concentration of caffeine and theophylline. Frequency and percentages were calculated for categorical data like nasal continuous positive airway pressure (CPAP), supplemental oxyen and intrventricular hemorrhage (IVH) grade I. Student t-test was applied in order to determine the significance of results. P value ≤ 0.05 was considered significant.

**Results:**

A total of 100 infants were enrolled in this study. This study was further divided into two equal groups by lottery method i.e. 50% in each, treated with Theophylline and Caffeine respectively. Mean apnea events/day in neonates administrated by theophylline was 1±0.1, 2±0.12, 2±1.1, 1±0.10 and 2±o.12 for 0, 1-3, 4-7, 8-14 and 15-21 days respectively. While, the mean apnea events/day in neonates administrated by Caffeine was 2±0.3, 1±0.22, 2±1.5, 1±0.13 and 2±0.14 for 0, 1-3, 4-7, 8-14 and 15-21 days respectively. The differences were statistically significant for 1-3 days and 4-7 days p<0.05 according to student t test.

**Conclusion:**

Results of our study revealed that caffeine being more effective than Theophylline for treating apnea of prematurity.

## INTRODUCTION

In the past, there have been reports of applicability of methylxanthines and theophylline being used for the cure of episodic apnea in premature infants till they complete a post-conceptional age of 34-35 weeks.^1^ As demonstrated by a literature review designed for the objective of prophylaxis or treatment of apnea of prematurity, some documentation regarding the use of theophylline or caffeine depicting some degree of altercation regarding the dosage regime^2^, the appropriate concentration of serum methylxanthine, the compulsion for drug monitoring, and the outcome of drug usage^3^, is still present.

A variety of studies have recommended a vast range of appropriate dosage regimen for caffeine (loading dose 3-40 mg/kg; maintenance dose 1.5–15 mg/kg/day caffeine-base). whereas the Safer reported dose as less than 80mg/L having 2-6 caffeine concentration, while 4 up to 93 mg/L is better tolerated and as less as 3–4 mg/L abates the apnea and accentuates the respiratory effort.^4^ Nonetheless, a high-loading dose of 25 mg/kg of caffeine, decrease the blood supply to cerebral and the intestinal areas in the pre-term neonates. It may result in neurologic complications akin to the transformation of astrocytogenesis which occurs after the post-natal exposure to low dose caffeine (less than 50 mg).^5^ Conversely, the degree of survival has been claimed to improve in apnea of prematurity (AOP) in very low birth weight (VLBW) infants upon the use of caffeine at optimal doses for the treatment.^6^ It does not bring about any neurologic incompetency at 18-21 months. It also decreases the likelihood of bronchopulmonary dysplasia. Adding further, the latest researches have established that if caffeine is used in standard doses (doses used in study), it does not necessitate the need for monitoring.^7^

Loading dose 4.4–9 mg/kg and maintenance dose 2–12 mg/kg/day have been used accomplishing plasma levels between 4–24 mg/L whereas levels as little as 1.3mg/dL are observed as efficacious.^8^ A strong recommendation of therapeutic monitoring has been made in order to get the therapeutic levels of theophylline between 5-12 mg/L. The prophylaxis of AOP by theophylline has not been successfully supported by previous studies. Neither of them has proclaimed the use of theophylline. Further research is recommended to determine the prophylaxis by methylxanthines. Being the inhibitors of adenosine receptors, both methylxanthines demonstrate the adverse effects and might as well poorly influence the pre-term development of the brain.^9^

Presently, it can be fairly described that caffeine is the approved methylxanthine in order to treat AOP in most of the neonatal care facilities. This observation is based on a limited amount of data.^10^ As such, the objective of the study was to make a comparison between theophylline and caffeine when used in standard doses in order to treat and prevent AOP in pre-term neonates to determine any differences in regards to (i) the episodes of apnea (ii) the relationship between serum concentration of methylxanthines and related apneic episodes and (iii) the requirement for monitoring of drug.

## METHODS

A randomized control study was conducted in Department of Pediatrics Medicine Govt. Khawaja Muhammad Safdar Medical College and Govt. Allama Iqbal Memorial Teaching Hospital, Sialkot over duration of one year August 2017 to August 2018 after approval from the ethics committee was obtained. An informed consent in the form of written document was also taken from the parents of each infant participating in the study.

The eligible candidates for the study included those infants born before 33 weeks of gestation. They were breathing spontaneously and were predicted to be administered with methylxanthine either therapeutically or prophylactically for AOP. Discontinuance of breathing for more than 20s or short but correlated with hypoxia (oxygen saturation <85%) or bradycardia (heart rate <100 bpm); was defined as Apnea.

After experiencing three episodes of apnea, infant was started upon the treatment, necessitating intense interventions with 24 hours, while if attacks of apnea were less than three in number, the infant was put on prophylaxis. All the participants were well maintained clinically, apart from their apneic events. Patients were divided into two groups (group A and B) by lottery method.

The elimination standard included congenital disorders, neuromuscular anomalies, perinatal asphyxia, IC hemorrhage, metabolic disorders, anemia, disseminated infection, severe GERD and previous introduction of methylxanthine.

A modification design that is computer propagated was utilized to describe the infants into the treatment groups Sigma Stat statistical package, Version 23. An authorized clinical pharmacist, un-involved with the research or the clinical management of infants otherwise, enforced the treatment assignment. The clinicians were not blinded due to use of different drug dosages and timings for blood sample.

After randomization, the infants were subjected to the use of theophylline (group 1) or caffeine (group 2) for the treatment of apneic episodes. The following optimal regime for the administration of theophylline was used. Theophylline (given intravenously in 30 minutes) loading doses 4.8 mg/kg (related to 6 mg/kg) and maintenance dose 2 mg/kg (up to 2.8 mg/kg) every 12 hour (related to 2.5–3.5 mg/kg) – caffeine citrate : loading dose 20 mg/kg (complementary to 10 mg/kg caffeine-base) given IV in 30 minutes and maintenance doses 5 mg/kg (up to 10 mg/kg) (consonant to 2.5–5 mg/kg caffeine-base) given IV in 30 minutes. (2.5–5 mg/kg caffeine-base) given once daily IV in 10 minutes. The therapy was carried on as far as the gestational age of 34 weeks or maybe shorter in case of transfer of infants to another hospital by sending physician with that child.

Blood sample of infants were withdrawn in order to determine the concentrations of caffeine and theophylline, as well as to determine the levels of caffeine in those candidates receiving caffeine citrate at the mentioned times of two hours after consummation of administration of loading dose and fifteen minutes prior to the introduction of maintenance doses on day three and day seven of therapy and every seven days thenceforth. Serum concentration of 5-12 mg/L was maintained and the dose of caffeine was modified upon signs of toxicity observed at the levels more than 50mg/L.

The observation of the infants was done before and during the methylxanthine treatment, with the help of a cardio respiratory monitor and a pulse oximeter. The other vitals recorded were heart rate, respiratory rate and oxygen saturation and the analysis of measurements was done by the administering physician in order to determine the episodes of apnea and it was examined by one of the authors after every 24 hours. The blinding was done for the author and the final data analyst for the prevention of bias. The alarm for tachycardia and bradycardia were set at 200 and 100 beats/minute respectively, while the oxygen saturation alarm was being set at 85%. At the bedside, a sheet for apnea nursing was maintained for all the infants upon whom the nurses were supposed to record the optimal intercessions for the corresponding events of apnea. The statistics of apnea did not alter the analysis of physician, done by nurses, to prevent under-estimation.

## RESULTS

One hundred (n=100) infants were enrolled in this study. This study was further divided into two equal groups i.e. 50% (n=50) in each, treated with Theophylline and Caffeine respectively. The mean gestational age of Theophylline group was 30.44±1.63 weeks. Mean gestational age of Caffeine group was 32.02±1.31 weeks. Supplemental O2 was observed as 24% (n=12) and 26% (n=13) for Theophylline group and Caffeine group respectively. Nasal CPAP was noted in 14% (n=7) and 10% (n=5) infants, for Theophylline group and Caffeine group respectively. While, IVH Grade-I was observed in 6% (n=3) and 12% (n=6) infants, for Theophylline group and Caffeine group respectively. The difference was statistically insignificant, except gestational age (p=0.000). (Table-I).

**Table-I:** Demographic Characteristics among the study groups.

Characteristics	Theophylline (n=50)	Caffeine (n=50)	P value
Gestational Age (weeks)	30.44±1.63	32.02±1.31	<0.001
Supplemental O2	24% (n=12)	26% (n=13)	0.817
Nasal CPAP	14% (n=7)	10% (n=5)	0.538
IVH grade I	6% (n=3)	12% (n=6)	0.295

The mean concentration at day 3, 7, 14 and 21 of Theophylline group was 6.62±1.69, 7.28±1.03, 6.93±0.93 and 6.44±1.21 respectively. The mean concentration at day 3, 7, 14 and 21 of Caffeine group was 13.29±2.50, 11.15±2.87, 12.19±2.82 and 11.06±2.07 respectively. The differences were statistically significant (p<0.005), according to student t test. (Table-II).

**Table-II:** Serum concentrations in infants receiving caffeine and theophylline for apnea of prematurity.

Characteristics	Theophylline (n=50)	Caffeine (n=50)	P value

	Mean concentration		
Day 3	6.62±1.69	13.29±2.50	<0.001
Day 7	7.28±1.03	11.15±2.87	<0.001
Day 14	6.93±0.93	12.19±2.82	<0.001
Day 21	6.44±1.21	11.06±2.07	<0.001

**Fig.1:**
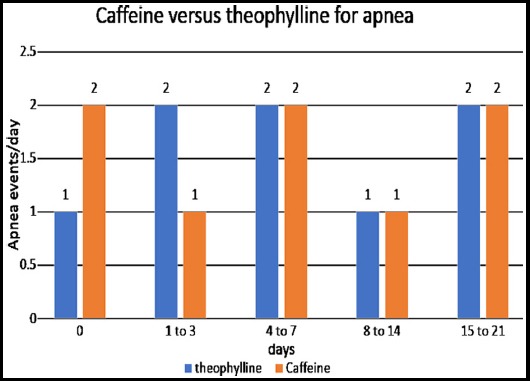


Mean apnea events/day in neonates administrated by theophylline was 1±0.1, 2±0.12, 2±1.1, 1±0.10 and 2±0.12 for 0, 1-3, 4-7, 8-14 and 15-21 days respectively. While, the mean apnea events/day in neonates administrated by Caffeine was 2±0.3, 1±0.22, 2±1.5, 1±0.13 and 2±0.14 for 0, 1-3, 4-7, 8-14 and 15-21 days respectively. (Fig.1).

## DISCUSSION

Study conducted by Natarajan G et al^11^ reported that if caffeine was used for the treatment of apnea in prematurity there was no need of additional monitoring for serum concentration of nitrogen, creatinine and enzyme level which is necessary to monitor in any other drug used for apnea in prematurity. He also reported that plasma concentrations of caffeine increased on high doses.

Maria S, et al^12^ also conducted a study on comparison of caffeine and Theophylline when used in apnea of prematurity and reported that caffeine is superior to Theophylline when used early stages of prematurity infants < 33 weeks. He also reported that both regimens not required extra monitoring in three weeks of prematurity. This study is comparable with our study.

In 2015 Jeong Ket al^13^ conducted a study and reported superiority of caffeine over theophylline in short term treatment of apnea in prematurity. He also reported that caffeine is effective and easy to administer. Shivakumar M et al^14^ compared caffeine with aminophylline and reported caffeine and aminophylline are equally effective.

In a study conducted by Mohammed Set al^15^ reported that caffeine can cause extubation failure in preterm ventilator patients and reduce the incidence of apnea in prematurity with minimum major side effects. Another study by Marcus CL, et al^16^ reported similar findings of apnea reduction.

Henderson-Smart DJ, et al^17^ reported caffeine and theophylline being equally effective for treatment of apnea but caffeine has more advantages over Theophylline. On other hand Theophylline have greater incidences of toxicity. These findings are also similar to our findings.

Steer PA, et al^18^ and Markantonis SL^19^ also reported similar findings and these studies are also comparable with our findings. According to this study caffeine is more safe and effective than any other drug used for apnea and prematurity.

## CONCLUSION

Results of our study revealed that caffeine is more effective than Theophylline in treatment of apnea of prematurity.

### Author’s Contribution

**AZ:** Conceived, designed and did statistical analysis & editing of manuscript.

**MH, KMS:** Did data collection and manuscript writing.

**ZAC:** Did review and final approval of manuscript.
